# Trauma‐focused imaginal exposure for auditory hallucinations: A case series

**DOI:** 10.1111/papt.12284

**Published:** 2020-05-21

**Authors:** Rachel M. Brand, Sarah Bendall, Amy Hardy, Susan L. Rossell, Neil Thomas

**Affiliations:** ^1^ Centre for Mental Health Swinburne University Hawthorn Victoria Australia; ^2^ Orygen: The National Centre of Excellence in Youth Mental Health Parkville Victoria Australia; ^3^ The Centre for Youth Mental Health The University of Melbourne Melbourne Victoria Australia; ^4^ Institute of Psychiatry, Psychology & Neuroscience King’s College London UK; ^5^ South London & Maudsley NHS Foundation Trust UK; ^6^ Psychiatry St Vincent's Hospital Melbourne Melbourne Victoria Australia

**Keywords:** auditory hallucinations, hearing voices, imaginal exposure, trauma, trauma‐focused

## Abstract

**Objective:**

There is mounting evidence that traumatic life events play a role in auditory hallucinations (AH). Theory suggests that some AH are decontextualized trauma memory intrusions. Exposure‐based trauma‐focused therapies that target trauma memory intrusions may therefore be a promising new treatment. We aimed to assess the feasibility and acceptability of a standard protocol trauma‐focused imaginal exposure intervention for trauma‐related AH and to provide initial proof of concept regarding potential effects on AH.

**Design:**

We conducted a single‐arm case series of a six session (90 min per session) imaginal exposure intervention for trauma‐related AH with assessments at baseline, post‐therapy, and one‐month follow‐up.

**Results:**

Fifteen people were recruited and eligible to start the intervention. Participants reported high levels of satisfaction; however, temporary distress and symptom exacerbation were common and contributed to discontinuation. There was a large reduction in AH severity at one‐month follow‐up (adjusted *d = *0.99), but individual response was highly variable. There were also large reductions in post‐traumatic stress disorder symptoms and the intrusiveness of the trauma memory.

**Conclusions:**

Imaginal exposure for trauma‐related AH is generally acceptable and may have large effects on AH severity for some people. However, temporary distress and symptom exacerbation are common and can lead to discontinuation. Low referral rates and uptake also suggest feasibility issues for standalone imaginal exposure for AH. The intervention may be more feasible and acceptable in the context of a broader trauma‐focused therapy. Well‐powered trials are needed to determine efficacy and factors that impact on acceptability and therapy response.

**Practitioner points:**

- Some AH can be understood as trauma memory intrusions that lack temporal and spatial contextualization and are therefore experienced without autonoetic awareness.
- Imaginal exposure to trauma memories associated with AH may be an effective intervention for some people.
- Temporary distress and symptom exacerbation may be common when using standard trauma‐focused imaginal exposure for AH. This can impact on the acceptability of the therapy and should be considered in future development and delivery.

## Background

Hearing a voice or noise in the absence of a corresponding external stimulus (variously termed ‘auditory hallucinations’, ‘hearing voices’, or ‘voice‐hearing’) is a common experience for people meeting a range of diagnostic criteria (Larøi *et al.*, 2012) and can lead to significant distress and disability. The current best‐practice psychological treatment for psychosis, cognitive behavioural therapy (CBT), has only shown small to moderate effects on auditory hallucinations (AH; van der Gaag, Valmaggia, & Smit, 2014). To date, therapies for AH have largely been derived from empirical evidence relating to the importance of beliefs about AH in maintaining AH‐related distress (Chadwick & Birchwood, 1994); however, there is growing evidence of other psychological processes that may be important in both the genesis and the maintenance of AH, providing an opportunity for improving treatments (Thomas *et al.*, 2014).

Post‐traumatic psychological sequelae are increasingly recognized to have involvement in psychotic symptoms, with mounting evidence that traumatic life events are associated with psychotic symptoms and that this relationship is causal (Bailey *et al.*, 2018; Kelleher *et al.*, 2013; Varese *et al.*, 2012). Theoretical models and empirical studies have implicated a range of post‐traumatic processes in the relationship between trauma and AH (Williams, Bucci, Berry, & Varese, 2018).

One strand of this literature has focused on the role of trauma memory intrusions. Trauma memory intrusions are of interest due to their phenomenological similarities with AH: both consisting of sensory experiences with no objective external stimulus, and experienced as involuntary and (often) to represent a current threat (Morrison, Frame, & Larkin, 2003). The content of AH has links to trauma content in approximately 50% of people with AH who have experienced trauma, with 12.5% of people having direct links, and 42.5% having thematic links (Hardy *et al.*, 2005). This suggests that a subset of AH may represent intrusions of traumatic memory material. Recently, Hardy (2017) has synthesized evidence in the area, theorizing that one pathway from trauma to AH may be related to aspects of trauma memory encoding and retrieval that increase intrusions of trauma memories. Shifts in information processing during traumatic events result in episodic memories that are fragmented, decontextualized, and predominantly sensory perceptual. This change in the encoding of episodic memory during traumatic events (termed *data‐driven processing*) is central in contemporary theories of post‐traumatic stress disorder (PTSD) and contributes to re‐experiencing symptoms (trauma memory intrusions and flashbacks; Brewin, Gregory, Lipton, & Burgess, 2010; Ehlers & Clark, 2000). Since psychosis is associated with impairments in spatial and temporal integration, it has been postulated that memories of traumatic events are more severely decontextualized in this group, leading to trauma memory intrusions that occur without autonoetic awareness and are therefore experienced as AH (Steel, Fowler, & Holmes, 2005). These AH are characterized by content that includes direct replays of aspects of traumatic events (Hardy, 2017). Other post‐traumatic psychological processes such as emotion regulation and negative post‐traumatic beliefs are implicated in shaping these trauma memory intrusion‐based AH and are also proposed as the basis of a second pathway from trauma to AH, likely characterized by thematic, but not direct content links between AH and traumatic events (Hardy, 2017).

Trauma memory intrusions are targeted in well‐evidenced *trauma‐focused* psychological interventions for PTSD, including prolonged exposure (PE), trauma‐focused CBT, and eye movement desensitization and reprocessing therapy (EMDR). Imaginal exposure, a central component of many of these interventions, encourages individuals to confront trauma memories in a safe environment. Proponents of PE hypothesize that fear habituation and reduction in negative post‐traumatic beliefs are key mechanisms of change (Cooper, Clifton, & Feeny, 2017); however, other models propose that imaginal exposure reduces intrusive trauma memories through elaboration and contextualization of the memory (Brewin *et al.*, 2010).

Despite people with psychosis historically being excluded from trials of trauma‐focused therapies, a recent trial has shown that a standard 8‐session PE or EMDR intervention for comorbid PTSD is safe and effective in this population (van den Berg *et al.*, 2015a, 2015b). A meta‐analysis also indicated promising secondary effects of trauma‐focused therapies on positive symptoms of psychosis (though this was based on a limited number of studies; Brand, McEnery, Rossell, Bendall, & Thomas, 2018). Exposure‐based trauma‐focused therapies have been highlighted as a particularly potent treatment component for treating post‐traumatic stress symptoms in psychosis (Hardy & van den Berg, 2017). Two recent case series have explored the effects of trauma‐focused psychological interventions specifically targeting AH (Keen, Hunter, & Peters, 2017; Paulik, Steel, & Arntz, 2019) with some promising findings. The treatment protocols in these studies did not have a central focus on trauma memory exposure, but did include elements of this (Keen *et al.* used a phase‐based approach incorporating reliving and rescripting, and Paulik *et al.* used imagery rescripting in which the rescript began before the distressing part of the memory).

Given the putative role of trauma memory processing and intrusions in some AH (particularly those that have direct content links to traumatic events) and indications that exposure‐based trauma‐focused therapies are particularly potent in targeting these processes, there is a strong rationale for their use in treating AH that have direct content links with traumatic events. In addition, exposure‐based trauma‐focused therapies reduce negative post‐traumatic beliefs, also giving rationale for their use in treating AH that are thematically related to traumatic events.

Despite the positive findings of the van den Berg *et al.* (2015a) trial for PTSD symptoms, there were no significant effects on AH (de Bont *et al.*, 2016). This may be due to the treatment targeting index traumas related to PTSD symptoms, rather than those specifically linked to AH. Further data are therefore needed on the potential of exposure‐based trauma‐focused therapies in treating trauma‐related AH. Some issues with the use of trauma‐focused approaches in psychosis have also been highlighted, with clinicians reporting reluctance in delivery due to concerns about symptom exacerbation and safety (Gairns, Alvarez‐Jimenez, Hulbert, McGorry, & Bendall, 2015) and young people with a first episode of psychosis receiving a trauma therapy reporting distress and psychotic symptom exacerbation (Tong, Simpson, Alvarez‐Jimenez, & Bendall, 2017). Exploring the feasibility, acceptability, and potential effects of exposure‐based trauma‐focused therapies when specifically treating AH is important to determine whether more comprehensive assessments of efficacy are justified and whether any adaptations to the therapy are required.

### Aims

We aimed to provide initial data on the feasibility and acceptability of using trauma‐focused imaginal exposure to treat trauma‐related AH. We also aimed to provide an initial proof of concept regarding the effects on AH severity, secondary symptoms (PTSD, delusions, depression, and anxiety), and postulated mechanisms of change (trauma memory intrusions, the nature of the trauma memory, and post‐traumatic cognitions).

## Methods

### Design

The Recall study was a single‐arm case series of a 6‐session imaginal exposure intervention for trauma‐related AH. An independent researcher assessed outcomes at post‐treatment and 1‐month follow‐up. The trial was prospectively registered as a pilot randomized controlled trial (ACTRN: 12616001503415), but was amended to a single‐arm study during the data collection phase due to slower recruitment than anticipated.

### Participants

We invited people attending a specialist voices clinic and people on an AH research participant registry to take part. We also promoted the study in local clinical services and consumer groups. Participants were required to (1) be aged 18–75; (2) have current AH (confirmed using item K6b of the MINI 7.02, Psychotic Disorders version, Sheehan et al*.*, 1998) that were frequent and persistent (present for more than 6 months and occurring at least twice a week); (3) report a history of PTSD criterion A traumatic events, childhood adversity, or significant bullying; (4) have made some conceptual links between their past adverse experiences and their AH (e.g., direct, content and indirect, thematic links, including emotional and temporal associations) and, for this reason, be motivated to undertake a trauma‐focused intervention; and (5) have a sufficient level of English language to participate in study requirements. Potential participants were excluded if (1) they had a recent (past month) or planned change in antipsychotic medication; (2) they had substance‐induced AH or current substance dependence issues that would interfere with participation in the study; (3) they demonstrated acute risk to themselves or others, defined by the presence of suicidal or homicidal thoughts with current intent; (4) their treating team reported that undertaking the study treatment would pose a serious risk to the safety of the participant or other people; or (5) they had an estimated IQ < 70 using the Wechsler Test of Adult Reading (Wechlser, 2001). We included experiences of interpersonal trauma that did not meet diagnostic criteria for PTSD (i.e., bullying, emotional abuse) because theory (Hardy, 2017) and research (Varese *et al.*, 2012) have linked these experiences to AH (the main focus of the study).

### Intervention

#### Imaginal exposure

The imaginal exposure intervention was delivered over 6 weekly 90‐minute sessions and was based on Foa’s PE manual (Foa, Hembree, & Rothbaum, 2007). Imaginal exposure in PE involves exposure to the trauma memory for a prolonged time through repeated recounting of the trauma narrative and listening to audio recordings. The main adaptation to the PE imaginal exposure protocol for the present study was in the first session; in addition to standard education regarding PTSD symptoms, trauma memory processing, and avoidance, time was also spent exploring links between traumatic events and AH content.

In contrast to standard PE, in which the targeted trauma memories are those that are most intrusive, therapy targeted those recognized as having a link with AH. These were identified through collaborative formulation in the baseline assessment and session 1 of treatment. The trauma memories identified as the most representative of distressing AH content, or that were most intrusive were prioritized for exposure work. Initial exposure sessions focused on a whole narrative of the traumatic event. Later exposure sessions were focused on memory ‘hot spots’ (i.e., those parts of the memory that seemed to represent the most distressing beliefs or emotions).

Therapy was delivered by a doctoral‐level, registered clinical psychologist (10 years post‐qualification) with experience in trauma‐focused therapies for PTSD and in psychological therapies for psychosis (RB) and supervised by a senior clinical psychologist with extensive experience in psychological interventions for AH (NT). Consultation regarding the therapy protocol and delivery was also provided by two specialists in the delivery of trauma‐focused interventions for people with psychosis (SB and AH). An overview of the intervention content is shown in Table 1. Adherence to the therapy protocol was assessed in 10% of sessions (randomly selected) by an independent researcher using a checklist of key elements for each session (see Appendix S1), with adherence found to be 95%.

**Table 1:** Intervention session content

Session	Content
1	Initial ratings of voice and memory intrusion frequency and distress Education regarding trauma and memory processing Discussion regarding the role of avoidance Review and elaboration of the client’s trauma‐voice link formulation established in the baseline assessment An explanation of the rationale for imaginal exposure Discussion of coping skills for managing any distress within and outside of sessions Out of session task: client to read a psycho‐education hand‐out regarding the nature of trauma memories and rationale for exposure
2–5	Voice and memory intrusion ratings Out of session task review Imaginal exposure exercise – full narrative, or hot spot work (20–40 min) Processing of cognitive and emotional aspects of the trauma memory through Socratic dialogue Out of session task: listen to imaginal exposure recording daily
6	Voice and memory intrusion ratings Out of session task review Imaginal exposure exercise – fully elaborated trauma narratives (20–40 min) Review of therapy progress and how client can continue to implement what they have learnt

### Measures

#### Baseline participant characteristics

We collected demographic information including age, gender, ethnicity, highest level of education, current psychiatric medication, and duration of AH.

Validated structured clinical interviews assessed current psychotic or mood disorders (MINI 7.02, psychotic disorders version, Sheehan *et al.*, 1998), borderline personality disorder (Structured Clinical Interview for DSM 5, First, Williams, Karg, & Spitzer, 2015), and PTSD (Clinician‐Administered PTSD Scale for DSM‐5, CAPS‐5, Weathers *et al.*, 2013a).

Trauma history was confirmed and described using the Life Events Checklist for DSM‐5 (Weathers *et al.*, 2013b), the Childhood Trauma Questionnaire (Bernstein and Fink, 1998), and an item from the Trauma History Questionnaire (Hooper *et al.*, 2011) assessing bullying.

At the end of the baseline interview, we rated whether there was a direct relationship between participant’s trauma exposure and AH content using criteria adapted from Hardy *et al.*(2005). This was done using up to three examples of participants’ most distressing AH content over the last week and details of the traumatic event from the CAPS‐5. A direct relationship was operationalized as AH content that included a literal correspondence to trauma content (i.e., AH content included exact words, phrases, or sounds heard at the time of the trauma).

### Feasibility and acceptability of the intervention

Our assessment of feasibility and acceptability of the intervention focused on uptake of and retention in the intervention. We recorded reasons for non‐consent, exclusion, or dropout throughout the study. Participants also completed the Client Satisfaction Questionnaire (Larsen, Attkisson, Hargreaves, & Nguyen, 1979) with an additional item measuring subjective improvement in AH.

### Primary effectiveness outcome: AH severity

We assessed AH severity using the Psychotic Symptoms Rating Scales – Auditory Hallucinations total score (PSYRATS‐AHS; Haddock, McCarron, Tarrier, & Faragher, 1999). Ecological momentary assessment (EMA) provided an additional measure of AH intensity and distress. Ecological momentary assessment involves measurements at intervals throughout an individual’s daily life and provides a sensitive, contextually valid measure that is less dependent on retrospective reporting. Participants were signalled to answer questions on a smartphone application, Movisens XS (https://xs.movisens.com), at 10 pseudo‐random time points each day (between the hours of 10 am and 8 pm) for 6 days (only at baseline and post‐treatment to reduce participant burden). The following questions were used: ‘Just before the beep went off I was hearing voices (that other people cannot hear)’, [if 2 or above] ‘This was distressing’ (rated on a scale of 1–7 where 1 = not at all, 4 = moderately and 7 = a lot). The items have previously been used in a psychosis population (Hartley, Haddock, Vasconcelos e Sa, Emsley, & Barrowclough, 2014). The EMA items were a subset of a longer battery of items being used in an adjoined study.

### Secondary effectiveness outcomes

Post‐traumatic stress disorder symptom severity was assessed using the CAPS‐5 (Weathers *et al.*, 2013a). The index trauma used for symptom severity ratings was the trauma identified as most related to the participant’s AH content. We assessed the severity of delusions using the Psychotic Symptom Rating Scales – Delusions Scale (PSYRATS‐DS; Haddock *et al.*, 1999) and depression and anxiety using the Depression Anxiety and Stress Scale–21 (DASS‐21; Lovibond & Lovibond, 1995).

### Mechanisms of change

Trauma memory intrusion intensity and distress were measured as part of the EMA schedule using the items: ‘Thinking about the traumatic or stressful event(s) we identified as related to your voices… since the last beep, memories of the event(s) came into my head when I did not want them to’, [if 2 or above] ‘This was distressing’. We used the Trauma Memory Questionnaire (TMQ; Halligan, Michael, Clark, & Ehlers, 2003) to assess the intrusiveness and disorganization of the trauma memory. We also assessed the extent to which memories were encoded in a sensory perceptual or a semantic (cognitive) form by analysing trauma narratives from participants’ first and final imaginal exposures using Linguistic Inquiry and Word Count (LIWC; Pennebaker, Boyd, Jordan, & Blackurn, 2015), calculating the percentage of words classified as ‘perceptual’ (e.g., see, hear) and ‘cognitive processes’ (e.g., cause, maybe, know). Negative post‐traumatic cognitions were assessed using the Posttraumatic Cognitions Inventory (PTCI; Foa, Ehlers, Clark, & Orsillo, 1999).

### Distress, symptom exacerbation, and adverse events

Participants who completed therapy rated how much distress they had experienced during their sessions. Serious adverse events were recorded throughout the study.

### Statistical analysis

Feasibility and acceptability results are reported descriptively. Our pre‐registered analysis plan for symptom and mechanism of change outcomes included significance testing using repeated‐measures analysis of variance and paired t‐tests; however, our final sample size meant that these tests would have been underpowered. In line with our aim to provide initial proof‐of‐concept data, we only examined effect sizes and 95% confidence intervals. Effect sizes between baseline and 1‐month follow‐up were calculated as *M*_pre_−*M*_post_/*SD*_pre_ and adjusted for unbiased *d* (Lakens, 2013). Effect sizes for non‐normal variables were calculated as *r* = *z*/√(*n*) (Rosenthal, 1991) and then converted to unbiased *d*.

Session‐by‐session ratings of AH and trauma memory intrusion frequency and distress were also collected and visually inspected to compare response trajectories of those with a direct trauma‐AH link and those without.

## Results

### Participant characteristics

Fifteen people were in the final study sample. Basic demographic and clinical characteristics of participants at baseline are shown in Table 2.

**Table 2:** Participant demographics (*n* = 15)

Age, *M* (*SD*)	43.79 (8.64)
Gender, *n* (%)	
Female	9 (60.00)
Male	5 (33.34)
Other	1 (6.67)
Ethnicity, *n* (%)	
Caucasian	13 (86.67)
Hispanic	1 (6.67)
Other	1 (6.67)
Highest level of education, *n* (%)	
Primary	1 (6.67)
Secondary	2 (13.33)
Tertiary	12 (80.00)
Index traumatic event type, *n* (%)	
Childhood sexual abuse	3 (20.00)
Childhood physical abuse	2 (13.34)
Childhood emotional abuse	4 (26.67)
Adulthood sexual abuse	5 (33.33)
Bullying	1 (6.67)
Workplace accident	1 (6.67)
Witnessing death of family member	1 (6.67)
Military trauma	1 (6.67)
Content link between AH and index trauma, *n* (%)	
Direct and thematic	3 (20.00)
Thematic	7 (46.67)
No link^a^	5 (33.33)
Primary diagnosis (MINI 7.02), *n* (%)	
Schizophrenia spectrum disorder	10 (66.67)
Mood disorder with psychotic features	4 (26.67)
Borderline personality disorder	1 (7.67)
Comorbid PTSD (CAPS 5), *n* (%)	6 (40.00)
Comorbid BPD (SCID 5), *n* (%)	3 (20.00)
Number of years had AH, *M* (*SD*)	19.17 (10.67)
Taking antipsychotic medication, *n* (%)	
Yes	11 (73.00)
No	3 (20.00)
Missing	1 (6.67)

AH = auditory hallucinations; BPD = borderline personality disorder; *M* = mean; *n* = number of participants; PTSD = post‐traumatic stress disorder; SD = standard deviation.

^a^ These participants reported temporal links between the trauma and AH onset, but had no content links.

### Uptake

Fifty‐six people were screened for eligibility over the recruitment period (November 2016 to January 2017 and February 2018 to December 2018). Twenty‐four people did not want to do a trauma‐focused therapy. Two declined participation on their treating clinician’s advice and two were not able to travel to the sessions. Ten people did not meet inclusion criteria: no trauma history (*n* = 5), no current AH (*n* = 4), intellectual disability (*n* = 1), and already receiving a trauma‐focused therapy (*n* = 1). Seventeen people were eligible and consented to take part. Two of these participants had to be withdrawn from the study at the baseline assessment stage (one due to substance use, and one on the advice of their treating team who had safety concerns).

The study was initially designed and registered as a pilot randomized controlled trial. We predicted a recruitment rate of two people per month, based on recruitment to previous AH therapy trials run locally. However, we recruited on average one person per month. As such, a ‘stop rule’ was initiated after eight months of recruitment, and the trial switched to a case series design.

### Retention

Fifteen people were enrolled to receive the study therapy. One participant was not contactable following the baseline assessment and did not begin the therapy. Of the 14 participants who did start the therapy, 11 completed all six sessions (all within eight weeks of their initial session). One participant ceased therapy after two sessions due to distress and symptom exacerbation but was willing to complete the follow‐up assessments. One participant ceased therapy after four sessions due to distress and symptom exacerbation and did not want to participate in follow‐up interviews. Another participant ceased therapy after two sessions due to an acute mental health inpatient admission (deemed unrelated to participation in the study by the participant and her treating psychiatrist) and was unable to complete follow‐up assessments. Twelve participants completed both EMA timepoints. Two participants responded to less than one third of EMA beeps at one or both timepoints and were excluded from the analysis (Delespaul, 1995). The 10 participants included in the analysis responded to an average of 46.1 beeps (*SD* 6.89) at baseline and 46.2 beeps (*SD* 7.83) at post‐treatment.

### Satisfaction

All participants who completed the satisfaction survey (*n* = 12) rated the quality of the treatment as excellent and that they were satisfied with the treatment (66.7% ‘very satisfied’, 33.3% ‘mostly satisfied). All participants reported that they would recommend the therapy to a friend who was in need of similar help (66.7% ‘yes definitely’, 33.3% ‘yes, I think so’) and that the therapy sessions helped them to deal more effectively with their problems (58.3% ‘yes, they helped’, 41.7% ‘yes, they helped a great deal’). Participants generally reported that their needs had been met by the therapy (58.3% ‘most of my needs have been met’, 25.0% ‘almost all of my needs have been met); however, 16.7% reported that only a few of their needs had been met. Over half of participants reported that their AH were improved following the therapy (50.0% ‘voices are better’, 8.3% ‘voices are much better’), but 25.0% reported no change in their AH and 8.3% that their AH were worse.

### Primary effectiveness outcome: AH severity

Mean reduction in the PSYRATS‐AHS was 3.5 points (95% CI −10.59, 3.59) at post‐treatment and 8.5 points (95% CI‐17.31, 0.31) at follow‐up (see Table 3), representing a large standardized effect size at this timepoint (adjusted *d* = 0.99). However, there was large variance in individual participant outcomes. Two participants had total remission from their AH at follow‐up.

**Table 3:** Outcomes measured at baseline, post‐treatment and follow‐up (*n* = 12)

Outcome	Baseline		Post		Follow‐up		Mean difference baseline‐post (95% CI)	Mean difference baseline‐follow‐up (95% CI)	ES
	*M*	*SD*	*M*	*SD*	*M*	*SD*			
PSYRATS‐AHS	29.58	7.96	26.08	11.04	21.08	12.94	−3.50 (−10.59, 3.59)	−8.50 (−17.31, 0.31)	0.99
CAPS‐5	26.75	14.89	15.17	11.38	11.92	10.54	−11.58 (−22.20, −0.96)	−14.83 (−25.97, −3.70)	0.93
PSYRATS‐D^a^	5	18.25	0.00	15.25	0.00	10.50	0.00 (−2.00, 0.00)	0.00 (−10.00, 1.00)	0.56
DASS Depression	10.25	6.14	8.25	5.50	8.00	5.51	−2.00 (−5.93, 1.93)	−2.25 (−5.87, 1.37)	0.34
DASS Anxiety	8.17	4.13	6.17	4.22	5.50	4.44	−2.00 (−5.56, 1.56)	−2.67 (−6.03, 0.70)	0.60
TMQ intrusiveness	2.48	1.04	1.66	1.15	1.29	0.92	−0.82 (−1.47, −0.18)	−1.19 (−1.78, −0.59)	1.06
TMQ disorganization	1.32	1.12	1.42	1.08	1.40	1.29	0.10 (−1.01, 0.81)	0.08 (−1.11, 0.94)	0.07
PTCI	136.33	36.20	114.75	45.72	107.83	50.89	−21.58 (−43.79, 0.62)	−28.50 (−54.87, −2.13)	0.73

Note

ES (adjusted *d*) reported for change between baseline and follow‐up.

CAPS‐5 = Clinician‐Administered PTSD Scale for DSM‐5; CI = confidence interval; DASS = Depression Anxiety and Stress Scale; ES = effect size; *M* = mean; PSYRATS‐AHS = Psychotic Symptom Rating Scales‐Auditory Hallucination Scale; PSYRATS‐DS = Psychotic Symptom Rating Scales‐Delusions Scale; PTCI = Posttraumatic Cognitions Inventory; *SD* = standard deviation; TMQ = Trauma Memory Questionnaire.

^a^ variable not normally distributed: median values (interquartile range), and median change (CI) reported.

EMA data showed reductions  of a small magnitude in mean AH intensity and medium magnitude for AH‐related distress (see Table 4).

**Table 4:** Outcomes measures at baseline and post‐treatment

Outcome	n	Baseline		Post		Mean difference baseline‐post (95% CI)	ES
		M	SD	M	SD		
AH intensity	10	3.50	1.89	3.34	1.94	−0.16 (−0.98, 0.66)	0.08
AH distress	10	4.37	0.74	3.78	1.52	−0.60 (−1.39, 0.19)	0.74
Intrusion intensity	10	2.65	1.44	2.45	1.39	−0.19 (−1.23, 0.85)	0.13
Intrusion distress	10	4.20	1.20	3.16	1.65	−1.04 (−2.34, 0.26)	0.81
Perceptual detail in trauma narrative^a^	11	3.45	0.98	2.67	1.19	−0.32 (−2.21, 0.47)	1.28
Cognitive processing in trauma narrative	11	10.04	3.83	10.63	4.39	0.58 (−1.76, 0.59)	0.14

Note

ES (adjusted *d*) reported for change between baseline and follow‐up.

AH = auditory hallucination; CI = confidence interval; ES = effect size; *n* = number of participants; *M* = mean; *SD* = standard deviation.

^a^ Variable not normally distributed: median values (interquartile range) and median change (CI) reported.

### Secondary effectiveness outcomes and mechanisms of change

Results for secondary outcomes and mechanisms of change are detailed in Tables 3 and 4. There was a large, consistent reduction in PTSD symptom severity at one‐month follow‐up (adjusted *d* = 0.93). EMA measures of trauma memory intrusions showed reductions in a small magnitude for mean intensity and a large magnitude for mean distress. Reductions in delusions, depression, and anxiety were small to medium.

There were medium to large changes in the intrusiveness of the trauma memory (adjusted *d* = 1.06) the level of perceptual detail in the trauma narrative (adjusted *d* = 1.28), and negative post‐traumatic cognitions (adjusted *d* = 0.73) at one‐month follow‐up. Reductions in the disorganization of the trauma memory and increases in cognitive processing in the trauma narrative were minimal.

Session‐by‐session ratings of AH and trauma memory intrusion frequency and distress are presented in Figures S1 and S2.

### Distress, symptom exacerbation, and adverse events

All participants who completed the satisfaction survey (*n* = 12) reported some level of distress in their sessions: 66.7% reported moderate distress that they felt able to manage, and 25.0% reported experiencing severe distress that they did not feel able to manage.

Three participants had mental health‐related inpatient admissions during the study. None of these admissions were deemed to be related to study participation (one admission prior to commencement of therapy for withdrawal from benzodiazepines, one admission between post‐treatment and follow‐up for electroconvulsive therapy, one admission for suicidal ideation that both the participant and psychiatrist reported to be unrelated to participation in the study). Of note, two participants did discontinue therapy and reported increased distress and exacerbation of PTSD and AH as a reason for this. One of these participants agreed to a follow‐up assessment, at which point their symptoms had returned to baseline levels.

## Discussion

The Recall study is the first study to examine the feasibility, acceptability, and potential effects of an exposure‐based trauma‐focused therapy, imaginal exposure, for trauma‐related AH. Our findings suggest promising effects for some AH, but also highlight some potential feasibility and acceptability issues in the use of standard exposure‐based trauma‐focused therapy specifically for trauma‐related AH.

### Is imaginal exposure a feasible treatment for trauma‐related AH?

The findings of the present study highlight some potential feasibility issues when delivering imaginal exposure specifically to treat trauma‐related AH. Referral and uptake rates for this study were low. The prevalence of traumatic events is known to be high in people with AH, and there is indication that many people identify their trauma history to be of importance in their AH (Corstens & Longden, 2013). We would therefore speculate that the reason for low referral and uptake does not reflect low demand for trauma therapies, but is perhaps related to perceptions of exposure‐based trauma‐focused therapies specifically. Indeed, clinician reluctance to undertake these therapies is well documented in PTSD treatment literature (Becker, Zayfert, & Anderson, 2004) and in psychosis (Gairns *et al.*, 2015). Further research into clinician and client perspectives of exposure‐based trauma‐focused therapies for AH is warranted. Because we were interested in examining exposure‐based trauma‐focused therapies as a standalone intervention, we required participants to have already made links between their AH and past trauma (so that exposure to identified trauma memories could commence in session 2). This may be another reason for low referral and uptake rates. Many clinical services traditionally have an emphasis on biomedical explanations of AH, meaning that many clinicians and clients in the mainstream mental health system may not have developed a trauma‐informed understanding of AH, possibly reducing the likelihood of uptake of a trial of this nature. Exposure‐based trauma‐focused interventions for AH may need to be delivered as a component of a broader trauma‐informed treatment in which links between AH and trauma can be formulated over time and trust in the rationale for exposure‐based trauma‐focused therapies developed.

### Is imaginal exposure an acceptable treatment for trauma‐related AH?

Participants who completed therapy reported high levels of satisfaction, despite the brief nature of the therapy and the use of trauma memory exposure early in treatment. The rate of dropout from therapy (26.7%) was in line with van den Berg *et al.* (2015a; 22.0%) and trauma‐focused therapies in PTSD populations (20‐27%; Hembree *et al.*, 2003). However, dropout was higher than that found in two recent case series examining different trauma‐focused therapies for AH. Paulik *et al.* (2019) used an imagery rescripting protocol and found a dropout rate of 8.3%. Keen *et al.* (2017) reported no therapy dropout from a phase‐based intervention in which stabilization preceded trauma‐focused work. This suggests that approaches that include a stabilization phase or that do not include direct exposure to the most distressing part of the trauma memory may be more acceptable when treating psychotic symptoms.

Importantly, participants who dropped out of the current therapy cited increased levels of distress and exacerbation of AH and PTSD symptoms that were too difficult to tolerate. Indeed, 25.0% of therapy completers also reported severe distress that they did not feel able to manage. This is in contrast with the large trial of PE and EMDR for comorbid PTSD in people with psychosis, which did not find exacerbation of psychotic or PTSD symptoms (van den Berg *et al.*, 2015a). This difference may be an artefact of the different focus of therapy in the present study (trauma‐related AH rather than PTSD), or to differences in participant characteristics or service contexts. Only 40% of participants in van den Berg’s (2015a) study had active AH, whereas all participants in this study had current AH. It is possible that distress and symptom exacerbation are more pertinent when working with trauma that is associated specifically with active psychotic symptoms. The tension between undertaking trauma memory exposure work and managing distress is inherent in all trauma‐focused therapies, and there has been much debate regarding the need for a ‘stabilization’ phase prior to memory exposure work, particularly in people with complex trauma histories (de Jongh *et al.*, 2016). The results here suggest that when treating trauma‐related AH, most people are able to tolerate trauma‐memory exposure work without stabilization, but a number of people may benefit from a stabilization phase prior to exposure work. It will be important to develop our understanding of clinical and contextual factors that influence the tolerability of exposure‐based trauma‐focused therapies for different people.

### What are the potential effects of imaginal exposure on trauma‐related AH?

We estimated a large effect of imaginal exposure on AH severity; however, individual participant changes were highly variable. With the small sample, confidence intervals around mean change scores were wide, so cannot rule out a null hypothesis of no effect. There was a small reduction in AH at post‐treatment and this reduction was of a larger magnitude at one‐month follow‐up, suggesting consolidation of therapy effects in the month following the end of therapy (possibly through continued processing of the trauma memory). Two clients experiencing complete remission from AH are notable given the chronicity of these experiences in our sample. Instances of complete remission from AH have been reported in other studies using trauma‐focused approaches for people with AH (van den Berg & van der Gaag, 2012; Paulik *et al.*, 2019) and may suggest that it is a particularly effective treatment for some people. We have provided a more in‐depth clinical reflection on factors that may have contributed to positive outcomes in two case illustrations from this study (Brand, Hardy, Bendall, & Thomas, 2019). Visual inspection of session‐by‐session data (Figures S1 and S2) raises the hypothesis that those with a direct link between their trauma and AH content may respond particularly well. This should be considered in future trials.

Ecological momentary assessment measures used as an additional measure of AH produced larger effect size estimates for AH‐related distress than for AH intensity. However, the large overlap between confidence intervals and fact that EMA data were only available at post‐treatment should be noted. In contrast, an exploratory examination of PSYRATS‐AHS subscales showed effects of a similar magnitude (medium–large) for AH frequency and distress at one‐month follow‐up (see Table S1).

The reductions in AH severity found in the current study are of a similar magnitude to those found for imagery rescripting (Paulik *et al.*, 2019) and phase‐based trauma‐focused therapy (Keen *et al.*, 2017). Although derived from small studies, this apparent equivalence in effectiveness suggests that clinician choice between imaginal exposure, imagery rescripting, and a phase‐based approach should be driven by time resources, clinical, and situational factors that may influence the tolerability of exposure work, and client preference.

### What are the likely mechanisms of action in imaginal exposure for trauma‐related AH?

Post‐traumatic stress symptoms showed large reductions over the course of treatment. The effects seen here are in line with those from previous studies (van den Berg *et al.*, 2015a) and suggest that participants received a sufficient ‘dose’ of imaginal exposure to act on the mechanisms of interest. The imaginal exposure did indeed have large effects on the intrusiveness of the trauma memory and perceptual detail in the trauma narrative, suggesting that it did impact on some aspects of the nature of the trauma memory, a key hypothesized mechanism of interest. However, there were minimal effects on the disorganization and cognitive processing of the memory. There were also medium effects on negative post‐traumatic beliefs, which, although not directly targeted, are in line with other studies of PE (Cooper *et al.*, 2017). As a small case series, these interpretations are speculative. The mediating effects of mechanisms of interest will need to be examined in well‐powered trials in the future.

### Strengths and limitations

To our knowledge, this is the first study to specifically examine the use of imaginal exposure for trauma‐related AH, providing novel data on the feasibility, acceptability, and potential effects of this approach. However, the small sample size limits conclusions regarding efficacy due to low power to detect effects. Similarly, the lack of a control group means that the specific effects of the intervention cannot be disentangled from natural changes in symptoms over time and from non‐specific therapy effects. The study also recruited a very specific group of participants (i.e., those who had already made links between their AH and their trauma history). The findings found here therefore provide a ‘proof of concept’ that this intervention *can* have some positive effects on AH, but a well‐powered randomized controlled trial is needed to definitively assess efficacy.

### Conclusions

Imaginal exposure for trauma‐related AH can have large effects on AH severity, but individual response is highly variable. Some people may find the process of exposure difficult to tolerate. Further research is needed to definitely assess efficacy and identify factors that influence therapy response and tolerability.

## Conflicts of interest

All other authors declare no conflict of interest

## Author contributions

Rachel Brand (Conceptualization; Data curation; Formal analysis; Funding acquisition; Investigation; Methodology; Project administration; Writing – original draft; Writing – review & editing) Sarah Bendall (Conceptualization; Methodology; Supervision; Writing – review & editing) Amy Hardy (Methodology; Supervision; Writing – review & editing) Susan Rossell (Conceptualization; Funding acquisition; Methodology; Project administration; Supervision; Writing – review & editing) Neil Thomas (Conceptualization; Formal analysis; Funding acquisition; Methodology; Project administration; Supervision; Writing – review & editing)

## Supporting information


**Table S1.** PSYRATS subscale scores at baseline, post treatment and follow‐up (*n *= 12).
**Figure S1.** Mean session‐by‐session ratings of AH and trauma memory intrusion frequency and distress (*n* = 11).
**Figure S2.** Mean session‐by‐session ratings of AH and trauma memory intrusion frequency and distress in those with a direct AH‐trauma content link (*n* = 3, a) and those without (*n* = 8, b).
**Appendix S1.** Therapy adherence checklist.

## Data Availability

The data that support the findings of this study are available from the corresponding author upon reasonable request.
