# Supplementary material for: Trauma‐focused imaginal exposure for auditory hallucinations: A case series
Source: Psychol Psychother. 2020 May 21;94(Suppl 2):e12284. doi: 10.1111/papt.12284 (PMC8246845; doi:10.1111/papt.12284)
Supplement: Supplementary file 1 — Table S1. PSYRATS subscale scores at baseline, post treatment and follow‐up (n = 12). Figure S1. Mean session‐by‐session ratings of AH and trauma memory intrusion frequency and distress (n = 11). Figure S2. Mean session‐by‐session ratings of AH and trauma memory intrusion frequency and distress in those with a direct AH‐trauma content link (n = 3, a) and those without (n = 8, b). Appendix S1. Therapy adherence checklist. [file PAPT-94-408-s001.docx]

**Supplementary material**

Supplementary Table 1.

| PSYRATS subscale scores at baseline, post treatment and follow-up (*n*=12) | | | | | | | | | |
| --- | --- | --- | --- | --- | --- | --- | --- | --- | --- |
|  | Baseline | | Post | | Follow-up | |  |  |  |
| Outcome | M | SD | M | SD | M | SD | Mean difference baseline-post (95% CI) | Mean difference baseline-follow-up (95% CI) | ES |
| PSYRATS-AHS distress | 14.50 | 4.78 | 13.08 | 6.08 | 9.92 | 7.33 | -1.42 (-4.46, 1.62) | -4.58 (-7.62, -1.54) | 0.89 |
| PSYRATS-AHS frequency | 7.67 | 2.78 | 6.25 | 3.11 | 5.67 | 4.03 | -1.42 (-3.19, -0.35) | -2.00 (-3.76, -0.23) | 0.67 |
| Note. ES (adjusted *d*) reported for change between baseline and follow-up. CI = confidence interval, ES = effect size, M = mean, SD = standard deviation. | | | | | | | | | |

Supplementary Figure 1. Mean session-by-session ratings of AH and trauma memory intrusion frequency and distress (n =11).


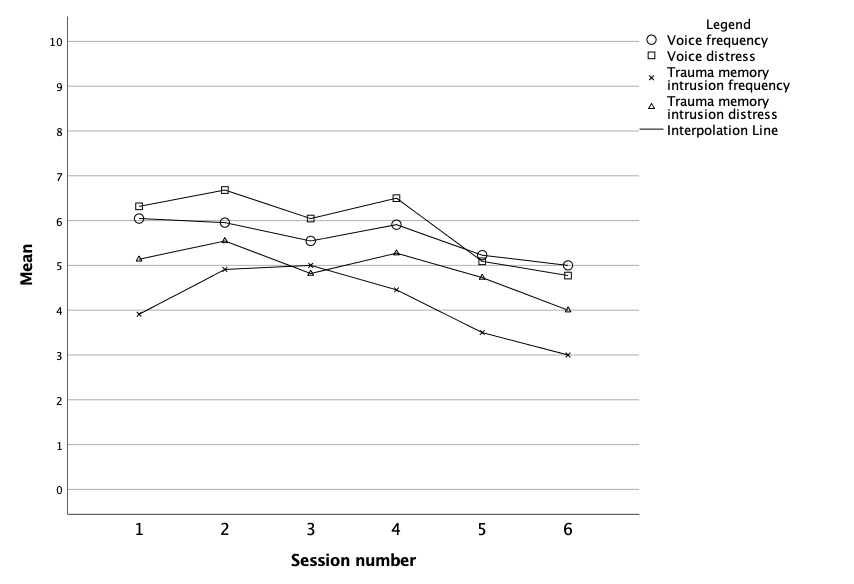


Supplementary Figure 2. Mean session-by-session ratings of AH and trauma memory intrusion frequency and distress in those with a direct AH-trauma content link (n=3, a) and those without (n=8, b).

a)


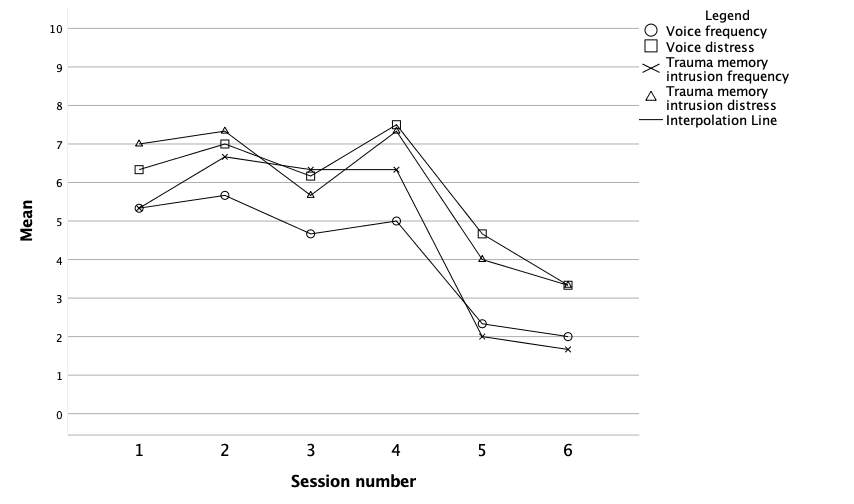


b)


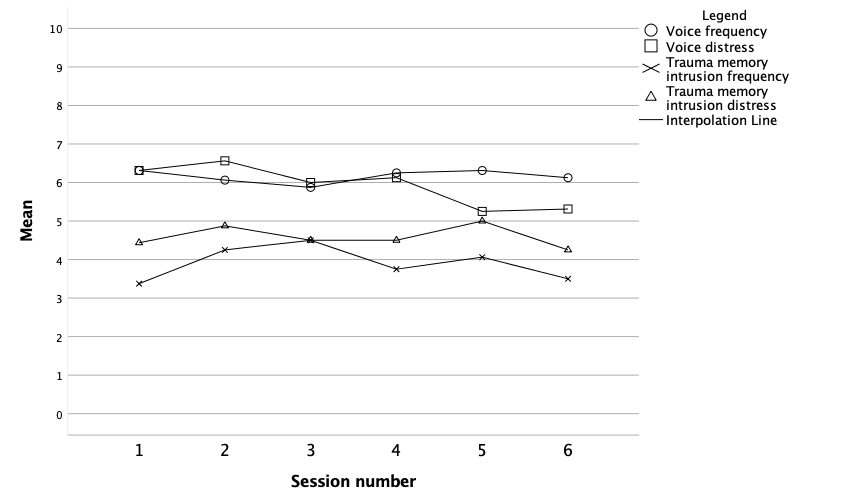


**Appendix S1. Therapy adherence checklist**

**Session 1**

| **Therapy element** | **YES** | **NO** |
| --- | --- | --- |
| 1. Therapist set an agenda for the session |  |  |
| 1. Therapist gave an overview of the treatment program (number of sessions, session length, out of session tasks, managing distress) |  |  |
| 1. Therapist provided an overview of memory processing during traumatic events and the consequences of this |  |  |
| 1. Therapist provided an overview of the role of avoidance in maintaining trauma-related symptoms |  |  |
| 1. Therapist provided an overview of common consequences of unprocessed memories and avoidance |  |  |
| 1. Therapist reviewed participant’s trauma-voice link formulation and elaborated in relation to psycho-education given |  |  |
| 1. Therapist described imaginal exposure |  |  |
| 1. Therapist outlined that confronting distressing memories facilitates processing of the trauma |  |  |
| 1. Therapist outlined that participant may get new insights or update old beliefs |  |  |
| 1. Therapist outlined that participant will learn that trauma memories are not dangerous |  |  |
| 1. Therapist assigned homework (read psycho-education handout) |  |  |

**Session 2**

| **Therapy element** | **YES** | **NO** |
| --- | --- | --- |
| 1. Therapist set an agenda for the session |  |  |
| 1. Therapist reviewed homework and gave feedback. If incomplete, therapist emphasised need for homework completion |  |  |
| 1. Therapist recapped the rationale for imaginal exposure, including introducing the filing cabinet metaphor |  |  |
| 1. Therapist introduced discomfort scale and worked with the client to create personalised ‘anchor points’ on the client’s scale. |  |  |
| 1. Therapist gave the participant instruction regarding how to conduct imaginal exposure (first person, present tense, eyes closed, keep going through distress, therapist will ask prompting questions) |  |  |
| 1. Therapist used appropriate re-enforcing comments during imaginal exposure |  |  |
| 1. Imaginal exposure lasted 20-40 minutes |  |  |
| 1. Therapist processed the imaginal exposure experience with the client |  |  |
| 1. Therapist assigned homework (listen to own audio of exposure daily/ cognitive therapy tasks) |  |  |

**Session 3-5**

| **Therapy element** | **YES** | **NO** |
| --- | --- | --- |
| 1. Therapist set an agenda for the session |  |  |
| 1. Therapist reviewed homework and gave feedback. If incomplete, therapist emphasised need for homework completion |  |  |
| 1. Therapist oriented the participant to the exposure planned for that session (including the rationale for, and identifying, hotspots if relevant) |  |  |
| 1. Therapist reminded the participant how to conduct imaginal exposure (first person, present tense, eyes closed, keep going through distress, therapist will ask prompting questions) |  |  |
| 1. Therapist used appropriate re-enforcing comments during imaginal exposure and elicited thoughts and feelings as appropriate |  |  |
| 1. Imaginal exposure lasted 20-40 minutes |  |  |
| 1. Therapist processed the imaginal exposure experience with the client |  |  |
| 1. Therapist assigned homework (listen to own audio of exposure daily/ cognitive therapy tasks) |  |  |

**Session 6**

| **Therapy element** | **YES** | **NO** |
| --- | --- | --- |
| 1. Therapist set an agenda for the session |  |  |
| 1. Therapist reviewed homework and gave feedback |  |  |
| 1. Therapist used appropriate re-enforcing comments during imaginal exposure |  |  |
| 1. Imaginal exposure lasted 20-30 minutes |  |  |
| 1. Therapist processed the imaginal exposure experience with the participant, focussing on reactions to and experience of imaginal exposure have changed |  |  |
| 1. Therapist spent time reviewing what participant has learnt and how this will apply to the future |  |  |
| 1. Therapist provided participant with positive feedback about work and progress |  |  |
